# Adaptation and implementation of clinical guidelines on maternal and newborn postnatal care in Iran: study protocol

**DOI:** 10.1186/s12978-023-01682-0

**Published:** 2023-09-12

**Authors:** Leila Abdoli Najmi, Sakineh Mohammad-Alizadeh-Charandabi, Shayesteh Jahanfar, Fatemeh abbasalizadeh, Haniyeh Salehi Poormehr, Mojgan Mirghafourvand

**Affiliations:** 1 Department of Midwifery, Faculty of Nursing and Midwifery, Tabriz University of Medical Sciences, Tabriz, IR Iran; 2 School of Public Health, Central Michigan University, Michigan, USA; 3 Women’s Reproductive Health Research Center, Tabriz University of Medical Sciences, Tabriz, Iran; 4 Research Center for Evidence-Based Medicine, Iranian EBM Center: A Joanna Briggs Institute Center of Excellence, Tabriz University of Medical Sciences, Tabriz, Iran; 5 Social Determinants of Health Research Center, Tabriz University of Medical Sciences, Tabriz, IR Iran

**Keywords:** WHO, Guideline, Adaptation, Postpartum care

## Abstract

**Background:**

According to World Health Organization (WHO), the postnatal care provision aims to provide care and treatment with the highest quality and the least intervention to obtain the best health and well-being for the family. The present study aims to adapt international guidelines for the clinical recommendations for the postpartum period and implement and determine its effectiveness.

**Methods/design:**

This study will be done in two phases. In the first phase, international clinical guidelines for mother and newborn postnatal care will be adapted. The second phase is a randomized controlled trial in which the adapted guideline recommendations will be implemented, and maternal and neonatal outcomes will be measured. The ADAPTE method for adaptation of clinical guidelines, is usedg in the first phase. A systematic review was conducted in the databases and clinical guidelines related to postpartum care were extracted according to the inclusion criteria. The quality of clinical guidelines was evaluated using the AGREE-II tool. The WHO clinical guideline obtained the highest evaluation score and was chosen as the main guideline, and the NICE clinical guideline, with a second higher evaluation score, was also used to fill some gaps in the WHO guideline. Based on the pre-determined questions, recommendations will be sent to the relevant experts and stakeholders for their evaluation. After the external evaluation and the finalization of the recommendations, the postpartum clinical guideline will be compiled and used in the second phase of the study. In the second phase, 272 women in the immediate postnatal stage of the maternity and postpartum ward of Taleghani and AL-Zahra Hospitals in Tabriz will be assigned into the intervention (receiving care based on adapted guidline recommendations) and control (receiving routine hospital care) groups uing individual stratified block randomization. At 6 weeks after birth, we will complete the Edinburgh postnatal depression scale, postpartum specific anxiety scale and Barkin index of maternal functioning (to assess the primary outcomes), as well as a maternal health problems checklist, infant care behavior, and violence assessment questionnaires (to asses the seconadary outcomes). Further, the maternal health problems checklist and the Edinburgh postnatal depression scale will be completed in the second week after birth. The data will be analyzed using an independent t-test and ANCOVA.

**Discussion:**

It is expected that the implementation of evidence-based clinical guidelines improves maternal and neonatal outcomes and experience of the postpartum period. The positive experience can also help to achieve Iran’s population policies and the need to increase childbearing in the country.

*Trial registration*: Iranian Registry of Clinical Trials (IRCT): IRCT20120718010324N76; Date of registration: 27/1/2023. URL: https://en.irct.ir/user/trial/66874/view; Date of first registration: 27/3/2023.

**Supplementary Information:**

The online version contains supplementary material available at 10.1186/s12978-023-01682-0.

## Plain language summary

The postpartum period, starting immediately after childbirth up to the first six weeks (42 days), is a significant and critical period in the lives of mothers and newborns, spouses, caregivers, and families. Maternal and newborn deaths and complications are high during the postpartum period. Postpartum care with high quality could reduce maternal and newborn deaths and complications, and makes the mother have a positive childbirth experience and a favourable attitude toward her next birth. World Health Organization (WHO) recommend providing medical care and treatment with the highest quality and the least intervention to obtain the best health and well-being conditions for the family. The present study aims to localize and use the international recommendations for the postpartum period in Iran. This study will be done in two phases. In the first phase, the international recommendations for mother and newborn care and postnatal well-being will be adapted according to our country's resources and conditions. In the second phase, the recommendations of adapted guideline will be implemented on 272 mothers and newborn in two groups in the maternity and postpartum ward of Taleghani and AL-zahra Hospitals in Tabriz, and we compare the result of using these recommendations and routine care on mothers' and newborns' well-being. In clinical guideline recommendations group, we will educate mothers, fathers and families and will share with them adapted recommendations, up to 6 weeks after birth and we will support them via the network. During the postpartum period we will assess mental and physical health problems of the mothers; counsel about breastfeeding, baby care, family planning, nutrition, activity, pain killer, bonding and emotional attachment, and prevention of postpartum depression; and any questions will be answered.

**Supplementary Information:**

The online version contains supplementary material available at 10.1186/s12978-023-01682-0.

## Background

The postpartum period, the first 6 weeks (42 days) after childbirth, is a significant and critical stage in the lives of mothers and neonates, spouses, caregivers, and families [1, 2]. Maternal and neonatal mortality and complications are high during this period, and up to 30% of maternal deaths occur after childbirth [3]. Recent global estimates of maternal mortality indicated that 287,000 women died during pregnancy and childbirth, mostly in low-income countries in 2020 [4]. The majority of deaths occur in the first 24 h after childbirth and up to one-fourth of these deaths can be attributed to postpartum hemorrhage, which is identified as the most common cause of maternal death after childbirth, and bleeding due to atony is the most important and increasing factor in developing countries [5].

The global strategy for the health of women, children, and adolescents has well-articulated the importance of postnatal care for mothers and newborns in ending preventable deaths and ensuring health and well-being by 2030. In particular, the third sustainable development goal (SDGs) is to reduce the neonatal and maternal mortality rate to less than 12 per 1000 live births and 70 per 100,000 live births by 2030, respectively [6].

Furthermore, the strategies designed to reduce maternal and neonatal mortality rates are confirmed and emphasized by the WHO. According to the WHO report, the burden of neonatal mortality is unacceptably high and despite the coverage of births by medical centers and maternity facilities and considering that the majority of births are performed by skilled individuals, 7700 infants (one every 13s) died everyday during the postpartum period in 2019; infants in the first month after birth with a global average of 17 deaths per 1000 live births were at high risk for death [7]. In general, three million neonatal deaths take place in the first 28 days of birth, about 1 million neonatal in the first 24 h of birth. WHO and UNICEF, in a joint statement, while emphasizing the importance of providing quality care, state: According to the evidence, the provision of postnatal care by qualified individuals leads to a 30–60% reduction in neonatal mortality in environments with few facilities [8]. Based on the WHO description, the postpartum care provision aims to provide medical care and treatment with the highest possible quality and the least intervention to obtain the best health and well-being conditions for the family. Postpartum care services are an essential part of the care continuity for mothers, newborns, and children to achieve SDGs in the field of reproductive and child health, reduce maternal mortality, and end preventable neonatal deaths [1].

Postpartum care with high quality makes the mother have a positive childbirth experience and a favourable attitude toward her next birth. Positive childbirth experiences can create a sense of control, independence, satisfaction, and confidence among mothers and affect the tendency to childbearing in the future [9]. However, the coverage and quality of postnatal care are relatively poor, especially in developing countries. The average coverage of routine postnatal care within two days after childbirth is about 71% for women and 64% for newborns, below the global targets for 2025 [10]. Furthermore, the utilization of postnatal care varies widely, especially in low- and middle-income countries where barriers to access are mainly based on socio-economic and rural conditions and low participation [11]. Unfortunately, postpartum care, despite being sensitive, is often given less attention in Iran by health care providers and midwives compared to the pregnancy period either qualitatively or quantitatively, and the average coverage of routine postnatal care is 74% in the country [12].

In Iran and Tabriz, postnatal care often receives less attention than the pregnancy period, resulting in inadequate coverage and low-quality care [13]. This study has the potential to enhance the utilization of postpartum care services, reduce mortality and morbidity rates, and improve postnatal well-being. By providing recommendations for clinical management, ensuring care continuity and consistency across wards and professions, and enhancing the quality of care, this study can influence policy-making and ultimately improve the health outcomes of mothers and newborns in Iran.

In the context of primary postnatal care of women and newborns, evidence-based guidelines can reduce mid- and long-term complications by improving care, influence the policy-making levels by providing recommendations in the field of clinical management, help to ensure the care continuity and uniformity across wards and care professions, and improve the quality of care [1]. Clinical guidelines, with the highest level of evidence in the evidence pyramid, are presented to change and improve the quality of care [14]. On the other hand, the implementation of clinical guidelines requires a comprehensive approach, including the consideration of local policies, resources, and contextual issues, which are localized based on the need for clinical guidelines specific to each region. The localization of clinical guidelines is an efficient alternative for designing clinical guidelines, especially in areas with limited resources [15, 16]. In this regard, International clinical guidelines based on the latest evidence, which can be used with any level of resources, in the form of comprehensive recommendations to improve the quality of essential and routine postpartum care.

Guideline adaptation is a potentially efficient alternative to de novo guideline development, especially in resource-constrained contexts [17]. In the field of postpartum care, high-quality guidelines have already been developed by international institutes using rigorous methodology [18]. Therefore, the present study aims to highlight the importance of adapting these clinical guidelines to local settings to reduce maternal and newborn mortality and morbidity and enhance postnatal well-being. The study will provide evidence-based recommendations for routine postpartum care that can be implemented in diverse settings, irrespective of available resources and facilities. The study will focus on adapting and implementing these clinical guidelines and assessing their effect on the outcomes of mothers and full-term newborns.

## Study aim

Adaptation and implementation of clinical guidelines on maternal and newborn postnatal care in Iran.

### Specific objectives

#### The first phase of the study

##### Main objective

To adapt clinical guidelines (the set of recommendations) for mother and newborn postnatal care in Iran.

#### The second phase of the study

##### Primary objectives

1. To compare mean score of maternal functioning at the sixth week after delivery between the intervention (receiving care based on adapted clinical guidelines) and control (receiving routine care) groups.
2. To compare mean score of postpartum depression in the second and sixth week after delivery between the intervention and control groups.
3. To compare mean score of postpartum anxiety in the sixth week after delivery between the intervention and control groups.

##### Secondary objectives

1. To compare mean score of infant care behavior in the sixth week after delivery between the intervention and control groups.
2. To compare frequency of postpartum health problems in the second and sixth weeks after delivery between the intervention and control groups.
3. To compare mean score of domestic violence in the sixth week after delivery between the intervention and control groups.

## Methods/design

### Study design

This study will be done in two phases. In the first phase, the clinical guidelines for mother and newborn postnatal care will be adapted. In the second phase, recommendations of the clinical guidelines will be implemented on mothers and newborns, and maternal and neonatal outcomes will be measured (Table 1). The phases of the study are explained in order:

**Table 1:** Postpartum care schedule of forms and procedures

Activity/assessment	Approximate time to complete	Enrolment Pre-study Screening/consent	Study period								
			Allocation	Post-allocation						Follow up 2-Weeks after delivery	Follow up 6-Weeks after delivery
Timepoint**		*-t*_*1*_	0	*t*_*1t*_	*t*_*2*_	*t*_*3*_	*t*_*4*_	*t*_*5*_	*t*_*6*_	*t*_*7*_	*t*_*8*_
Pre-study Screening/consent	3 Minute										
Eligibility screen	3 Minutes	X									
Informed consent	4 Minutes	X									
Allocation	3 Minutes		X								
Demographics Questionnaire	4 Minutes		X								
Checklist of maternal health problems Postpartum depression	5 Minutes		X							X	X
	5 Minutes		X							X	X
Care and education based on the adapted guideline in hospital	15 Minutes		X								
Education and support via network 6 weeks after delivery	NA		X	–––––––––––––––––––							
Postpartum anxiety	5 Minutes										X
Infant care behavior	5 Minutes									X	X
Maternal Functioning	5 Minutes										X
Violence questionnaire	2 Minutes										X

## First phase

### Study design

To avoid duplicating efforts, make cost-effective use of available resources, and facilitate customization of guidelines to reflect local contexts, we opted to adapt rather than develop the guideline from scratch. Based on the results of the study in some cites in Iran, the rate of cesarean section is high (62–72%) [19]. Therefore, we need to develop a comprehensive clinical guideline that provide recommendations for vaginal childbirth and caesarean section, the ADAPTE method was used to the adaptation of clinical guidelines [20].

### Procedure

The ADAPTE process is used in the present study (Table 2). It is a framework for guideline adaptation, which consists of 3 phases and 24 steps. The three phases of ADAPTE process including planning and set-up, adaptation, and development of a final product. The set-up phase is the preparation for the adaptation process [21].

**Table 2:** Adapting the guidelines using the ADAPTE process

Step	Activity	Result
*Phase I: set up*		
1	Establish a resource team	The research team (steering team), consisting of five members, was responsible for developing the terms of reference and compiling a list of potential members for the working group. Based on this list, the steering team extended invitations to individuals to join the group
2	Determine criteria for selection and select a topic using criteria	Postpartum was chosen as the focus because there is currently no national guideline for postpartum care in the country, and there are variations in the standards of care being provided that needed to be updated
3	Check if adaptation is feasible	Internationally, evidence-based guidelines for postpartum care were already in use, and there was considerable interest from the Ministry of Health, clinicians, and other stakeholders to develop similar guidelines
4	Identify necessary resources and skills	There was a high level of commitment among the members of the working group, and all the required areas of expertise were available, including obstetrics, reproductive health, midwifery, nurse, policy development, and neonatology
5	Complete tasks of the set-up phase	Members of the group decided to function as a working group coordinated by the Research Vice-Chancellor of the Faculty of Nursing and Midwifery
6	Write the plan for adaptation	A timeline for completion was established, additional resource persons to be included were identified. Task allocation among the members was also agreed upon
*Phase II adaptation*		
7	Determine and clarify the question	A PIPOH summary (Table 4) was prepared, and the areas of interest for postpartum standards of care were identified as assessment, prevention, screening, and education during the postpartum period
8	Search for guidelines and other relevantocumentation	The steering team searched for relevant postpartum guidelines
9	Screen the retrieved guidelines and record their characteristics and content	The recommendations of the guidelines for assesment, prevention, screening, and education of postpartum were reviewed, extracted, and compiled in summary tables
10	Eliminate a large number of the retrieved guidelines using the AGREE instrument	The rigor dimension of the AGREE II tool was utilized to eliminate guidelines that did not meet the stipulated criteria
11	Assess the quality of the guideline	The AGREE II instrument was used to scrutinize the quality of the guidelines
12	Assess the currency of the guideline	The retrieved guidelines were sufficiently up-to-date, and no new evidence was identified
13	Assess the content of the guideline	Recommendations for assessment, prevention, screening, and education were examined, and while there were some differences in the content of the guideline, they were not found to be contradictory
14	Assess the consistency of the guideline	There was clear consistency between the evidence from systematic reviews, the interpretation of the evidence, and the recommendations in the guidelines in all the areas of interest
15	Assess the acceptability and applicability of the recommendations	Care was taken to ensure the recommendations are not in conflict with other local guidelines and to appraise the implications of the guidelines on health service delivery
16	Review assessments	The results of the assessment of the guidelines were discussed in meetings of the working group
17	Select among guidelines and recommendations to create an adapted guideline	The WHO and NICE guidelines were the main guideline used because the recommendations compared well with the other guidelines and the practice-based recommendations were well-stated
18	Prepare a draft of the adapted guideline	The member of the research team compiled the results of the deliberations and wrote the draft guideline document
*Phase III finalization*		
19	Seek feedback on the draft guideline from those who would be using it	A draft of the guideline will be circulated for comments to the working group members, as well as obstetricians, reproductive health specialists, midwives, and other relevant stakeholders
20	Consult with endorsement bodies	The guidelines will be adopted by the Ministry of Health
21	Consult with developers of guidelines used as sources	Decisions about the recommendations will be made based on the available resources and facilities, as well as expert opinions regarding clinical benefits and localization capabilities
22	Acknowledge source documents	The key guidelines used for developing the local protocol will be cited
23	Plan for aftercare of the adapted guideline	A review date has been scheduled for five years from now, and monitoring indicators have been identified. The guidelines will be distributed in both electronic and print copies
24	Produce a final document of the guideline and other outputs	The following additional outputs will be produced (along with the guideline): posters and brochures for patient information, workshop slides for training health workers, Clinical Manual for Maternity Care, and quality assurance guidelines

In the set up phase, the stakeholders' recognition of the need for postpartum guideline prompted the research team to form a steering team consisting of five members, and working team including 20 specialists and experts in the related subjects, methodologists, and experts in the field of maternal and neonatal health were selected and invited to the group. After specifying the desired areas (assesment, prevention, screening, and education) in the field of maternal care during the postpartum period, several issues have been identified as important due to their association with the lowest median national coverage of interventions on the continuum of maternal and child healthcare. These issues include low education, poverty, limited access to healthcare facilities, and variation in care delivery at different locations and settings. These factors can lead to disparities in care and outcomes for women and their infants. At this step, the feasibility of adaptation of the clinical guidelines was investigated and approved by the group. The target population of the clinical guideline includes all mothers and their babies, fathers, families and healthcare providers (midwives, physicians, nurses, etc.). The main area in which the guideline should be used includes the primary (health care settings), the secondery (public hospitals), and the tertiary level (specialized and super-specialized hospitals) and community. The search for the guidelines was done through a systematic search of the databases and specific websites of the clinical guidelines including: Guidelines International Network (GIN), National Guideline Clearinghouse (NGC), World Health Organization (WHO), Scottish Intercollegiate Guidelines Network (SIGN), National Institute for Health and Clinical Excellence (NICE), Clinical Practice Guidelines (CPG Infobase), Trip database, Cochrane Library, and PubMed. The search was limited to December 2010 to December 2022. For example, the search strategy for PubMed database is provided in the Additional file 1.

### Inclusion and exclusion criteria of clinical guidelines

The inclusion criteria of clinical guidelines are the evaluation and approval of the guidelines by Appraisal of Guidelines for Research and Evaluation (AGREE II) checklist, English language, appropriate organizing, possibilty of access to the full text of the guideline, and being essential postnatal care as the scope of the guideline. The exclusion criteria for guidelines include the different target groups of clinical guidelines and the difference in the topic of clinical guidelines and publication before December 2010. The inclusion criteria for adaptation team members are methodological expertise, clinical expertise, and willingness to attend in the sessions. The data are collected through the searched guidelines and the comments of adaptation team members.

### Validity and reliability of AGREE II

(AGREE II) checklist was used to evaluate the extracted clinical guidelines. This 23-item checklist includes six areas and each section deals with one aspect of guideline quality [22]. The authentic translation of the AGREE II checklist was prepared through the cooperation agreement between the Tehran University of Medical Sciences and the Health Deputy of the Ministry of Health and Medical Education in the field of the National program for clinical guideline development in 2016. Researchers can use AGREE II checklist as a reliable reference to check the quality of clinical guidelines to the adaptation of clinical guidelines [23].

In the adaptation phase, designing the kind of evidence required to analyze and offer recommendations in the field of postpartum care was proposed under the title of designing answerable questions. First, the research team identified 22 clinical questions (see Table 3) in the areas of maternal and newborn assessment, intervention, education, and prevention in the functional field of postpartum care and reviewed and confirmed with the opinion of relevant experts. Considering that the research team aimed to adapt clinical guidelines in the field of maternal and neonatal care after delivery, a systematic search was conducted to search for clinical guidelines and other related documents. At this stage, the inclusion and exclusion criteria of clinical guidelines and other documents were determined. Then, PIPOH (Table 4) was designed and accordingly, appropriate keywords, including ‘postpartum care’, ‘postpartum period’, ‘postnatal period’, ‘postnatal care’, ‘puerperium’, and ‘postpartum programs’ were selected and the keywords of ‘guideline’, ‘guidance’, ‘recommendation’, ‘consensus’, ‘best practice’, ‘statment’ were added to the search databases of guidelines. Based on the aforementioned databases, 22 guidelines were retrieved. After initially reviewing the retrieved guidelines by the search team, 6 duplicates, and 6 ineligible guidelines were eliminated, 10 guidelines were selected for appraisal, then 5 guidelines were removed based on the exclusion criteria. Finally, The five guidelines were selected (Table 5) for the second phase of the adaptation process and were evaluated and criticized in terms of quality, currency, content, consistency (evaluation of search strategy, selection and interpretation of evidence) and acceptability (applicability of recommendations). The quality of clinical guidelines was evaluated and graded by two reviewers (LAN and MM) independently under supervision of third person (SMAC), by using the AGREE II Instrument for the evaluation of each guideline (Table 6). The latest version of clinical guidelines was selected in terms of currency. The stability was evaluated by the research team and the content and applicability of the recommendations were assessed by the working team.

**Table 3:** Maternal and Neonatal Health Questions

1	What is the recommended standardized procedures for assesment of women after vaginal and caesarean delivery?
2	What are recommended methods that can be used to alleviate pain after delivery, for both vaginal and caesarean section births?
3	What are recommended effective methods for relieving perineal pain ?
4	What are recommended effective methods for relieving pain and promoting healing at the caesarean section site?
5	How can breast congestion be alleviated effectively?
6	Which methods are recommended for preventing and healing nipple sores?
7	? Should everyone be screened for postpartum depression? Which method is recommended
8	? Which women? What supplements do women need to receive in the postpartum period
9	When is the recommended time to start physical activities after vaginal delivery and cesarean section?
10	When is the recommended time for discharge after vaginal delivery or cesarean section?
11	When are appropriate times for a mother to visit health centers to receive postpartum care?
12	Which items should be evaluted as part of the assessment of postpartum women in health centers?
13	Are pelvic floor exercises recommended after natural birth and cesarean section, and since when?
14	What are the most important psycho-social support methods for mothers to prevent postpartum depression?
15	What are symptoms and signs of illness in babies and mothers?
16	Which assessments are recommended for a healthy newborn in the postpartum department?
17	Is it necessary to bathe the baby in the hospital?
18	Which screenings are recommended for all newborns?
19	What are the recommendations for promoting bonding and emotional attachment between parents and their child?
20	and prevent of postpartum depression? What are the recommendations to improve the postpartum experience
21	What are the ways to promote the participation of fathers in the postpartum period?
22	What are the recommendations to provide informations and support mothers in breastfeeding?

**Table 4:** PIPOH summary of the clinical questions

P	Population	All postpartum women and newborn without complications
I	Intervention	Assessment, Screening, prevention, and education in the postpartum period
P	Professions (target users)	Primary care workers, midwives, physician, nurses, policy-makers
O	Outcomes	Health promotion of all postpartum women and newborn without complications
H	Health care setting	Community, Primary, Secondary, and Tertiary level health care settings

**Table 5:** Characteristics of included guidelines

Country	Guideline group	Abbreviated name	Title	Year
Canada	Public Health Agency of Canada	CMA	Postpartum care [35]	2020
USA	American College of Obstetricians and Gynecologist	ACOG	Optimizing postpartum care. ACOG Committee Opinion No 736 [36]	2018
France	French College of Gynecologists and Obstetricians	CNGOF	Postpartum practice: guidelines for clinical practice from the French College of Gynaecologists and Obstetricians [37]	2016
International	World Health Organization	WHO	WHO recommendations on maternal and newborn care for a positive postnatal experience [1]	2022
UK	National Institute for Health and Care Excellence	NICE_194_	Postnatal care [38]	2021

**Table 6:** AGREE II domain scores for the included guidelines

Organization	Scope and purpose Percent	Stakeholder involvement Percent	Rigor of development Percent	Clarity of presentation Percent	Applicability Percent	Editorial independence Percent	Overall guideline assessment Percent
WHO (2022)	100	100	100	100	97.9	100	Strongly recommended
NICE (2021)	100	100	95	95	95	95	Strongly recommended
CMA (2020)	57	40	20	80	20	0	Recommended with alterations
ACOG (2018)	47	60	30	52	18	60	Recommended with alterations
CNGOF (2018)	45	45	55	45	15	57	Recommended with alterations

**Fig. 1:**
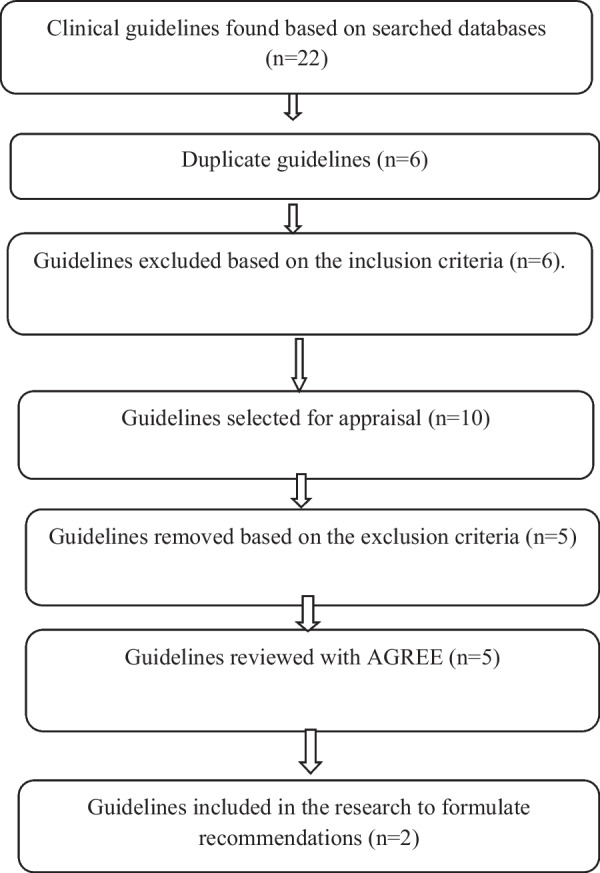
The search and selection guidelines used for the analysis

Among the five evaluated guidelines, two clinical guidelines got the highest evaluation scores (Fig. 1). According to the evaluation-related consensus of the research team, 2022 WHO clinical guideline for mother and child care and postnatal well-being was selected for adaptation. The recommendations of the 2021 NICE clinical guideline were applied according to the desired research questions and research setting. In the third phase of adaptation (finalization of recommendations), the recommendations of WHO and NICE will be sent as a form to obtain the opinions of experts in terms of acceptability and applicability. In the finalization phase, delphi method will be used to gather experts' opinions. The final recommendations will be written after their evaluation by external observers. Decisions about the recommendations will be made based on the available resources and facilities, as well as expert opinions regarding clinical benefits and localization capabilities. The guideline will be adopted by the Ministry of Health. A review date will be scheduled for five years from now, and monitoring indicators will be identified. The guideline will be regularly monitored and updated as necessary to ensure that it continue to reflect current evidence-based practices. Both electronic and print copies of the guideline will be distributed to relevant stakeholders for easy access.

In the second phase, the recommendations of the clinical guideline will be implemented in the form of a clinical trial and maternal and neonatal outcomes will be measured.

## The second phase

### Study design

**Fig. 2:**
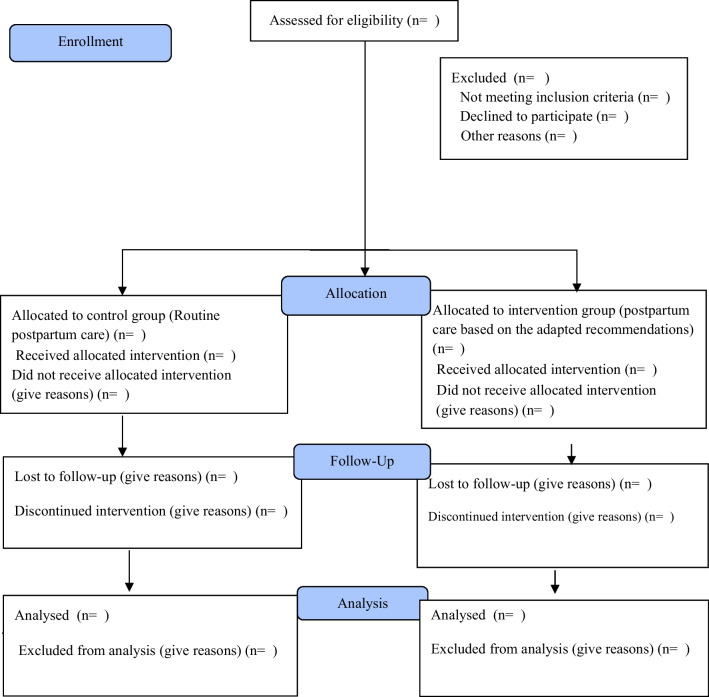
Flow of participants

The second phase of the current research is a randomized controlled trial (Fig. 2) and the study population is women who have given birth and hospitalized in the maternity and postnatal wards of Taleghani and AL-Zahra hospitals in Tabriz.

### Inclusion criteria

The inclusion criteria are mothers with term babies, singleton birth, and a secondary or higher level of education.

### Exclusion criteria

The exclusion criteria include the occurrence of trauma during the last three months, including the death of loved ones, divorce, etc.; obtaining a depression score of 13 and above based on the postpartum depression questionnaire; the presence of chronic diseases in the mother, including cardiovascular, chronic blood pressure, diabetes, etc.; mothers with babies hospitalized in the NICU.

### Sample size and sampling method

The sample size was calculated based on the variables of maternal functioning, specific postpartum anxiety, and postpartum depression using G-Power software. According to the study of Gholizadeh Shamasbi et al. [24], concerning the maternal functioning variable and considering M_1_ = 97.4, M_2_ = 116.9, with the assumption of a 20% increase due to the intervention, SD_1_ = SD_2_ = 12.9, Two-sided α = 0.05, and Power = 95%, the sample size was calculated 13 per group. Further, based on the study results of Mirghfourvand et al. [25] on specific postpartum anxiety variables and considering M_1_ = 14.28, M_2_ = 11.42, with the assumption of 20% reduction due to the intervention, SD_1_ = SD_2_ = 8, Two-sided α = 0.05, and Power = 80%, the sample size was calculated to be 124 participants. According to the data available in the study of Ahmadi et al. [26] regarding postpartum depression variable and considering M_1_ = 13.5, M_2_ = 10.8 (assuming 20% reduction due to the intervention), SD_1_ = SD_2_ = 5.8, Two-sided α = 0.05, and Power = 95%, the sample size was obtained 121 per group. Given that the sample size calculated based on the postpartum anxiety variable was more, the final sample size was considered 136 in each group (considering 10% attrition).

### Sampling

Eligible women will be selected from postnatal wards of Taleghani and AL-Zahra hospitals in Tabriz using the conventional sampling method. First, full explanations about the study and its objectives and method will be provided to the women and written informed consent will be obtained from the women willing to participate in the study. The socio-demographic, obstetric and neonatal characteristics and postpartum depression questionnaire will be completed. Then, after the random allocation, the intervention program will be implemented for the intervention group and routin care for the control group.The mothers and their babies will be followed up until 6 weeks after birth. The questionnaires of maternal health problems will be completed in the second and sixth weeks after birth; and questionnaires of infant care behavior postpartum depression, specific postpartum anxiety, and maternal functioning, and violence in the sixth week after delivery in the intervention and control groups (Table 1).

### Randomization method and allocation concealment

A computer program will be used to create a random allocation sequence. The participants will be assigned into the intervention and control groups with a ratio of 1:1 using the stratified block randomization method (stratified based on parity and type of delivery) with a randomly varied block sizes of 4 and 6. For allocation concealment, the type of intervention will be written on paper and placed in consecutively numbered opaque sealed envelopes. After obtaining the written informed consent form, the relevant envelope will be opened and the intervention will be implemented. Due to the nature of the intervention, the researcher and participants are not blinded. To blind the data collectors, the questionnaires related to the postpartum stage will be completed by the research assistant.

### Intervention

After assigning the participants to the intervention and control groups, the socio-demographic and obstetric questionnaire will be completed. The adapted recommendations of the clinical guidelines will be implemented in the intervention group. The recommendations for mother and newborn care in the maternity and postnatal wards will be provided to mothers by the researcher. The educational recommendations concerning maternal self-care and baby care, nutrition, mobility, danger signs of mother and baby, breastfeeding, baby massage, bilirubin screening in newborns, contraceptive methods, use of supplements, etc. will be presented in the postnatal ward through face-to-face education and on the virtual network. In the same vein, a 10–15 min education session will be held for mothers based on the recommendations of the clinical guideline in the education room of the postnatal ward. The content of the recommendations will be sent to mothers and fathers in the form of simple text and a short educational video on the virtual network. Rubika, one of the Iranian social networks which is easy to use and widely accessible, will be utilized for providing virtual training. The researcher will be responsive for all postnatal problems for up to six weeks and 24 h a day,and provide solutions based on the recommendations of the clinical guideline on the virtual network to mothers and fathers in the intervention group. Mothers in the control group will receive routine postnatal education.

### Scales and data collection

In the present study, the data will be collected using a socio-demographic, obstetric and neonatal characteristics questionnaire, maternal health problems checklist, Edinburgh postnatal depression scale (EPDS), postpartum specific anxiety scale (PSAS), infant care behavior questionnaire, Barkin index of maternal functioning (BIMF), and violence (HITS = Hurt, Insult, Threaten, and Scream), which will be completed through interview and observation.

### Socio-demographic and obstetric and neonatal characteristics questionnaire

This questionnaire includes the variables of demographic and social charactristics (age, education level, occupation, religion, ethnicity, marital status, residence status, household income, maternal charactristics (number of pregnancies, abortions, gestational age, birth and breastfeeding problems, participation in childbirth preparation classes, delivery by a doctor or midwife, having tear or episiotomy, type of analgesia for birth, type of analgesia, indication for cesarean section), neonatal characteristics (weight, height, head circumference, position and frequency of breastfeeding, and neonatal jaundice).

### Checklist of maternal health problems

The checklist of maternal health problems includes items related to constipation, hemorrhoids, anal ulcers, urinary tract infection, cold or fever, back pain, leg cramps, joint pain, abdominal pain, dizziness, headache, mouth inflammation and bleeding gums, breast inflammation and redness, breast pain, nipple fissure (nipple wound), mastitis, episiotomy and cesarean incision pain and infection, heavy bleeding for more than a week, sleep problems, urinary incontinence, weakness or fatigue, sadness and discomfort, and abnormal postpartum discharge. This questionnaire will be designed by the research team and its content and face validity will be checked and confirmed by 10 faculty members of Tabriz University of Medical Sciences.

### Edinburgh postnatal depression scale (EPDS)

This scale, developed by Cox et al. (1987), is used to measure depression during pregnancy and the postnatal period. EPDS consists of 10 items on a 4-point Likert scale, rating from 0 to 3 based on the severity of the symptoms. The total score range varies between 0 and 30. Some items (1, 2, and 4) are arranged from low to high severity and the others are from high to low severity. The validity of EPDS was calculated to be 0.78 by determining the simultaneous correlation coefficient of the Edinburgh scale and the Beck depression scale and its reliability was estimated to be 0.75 using Cronbach's alpha and halving method [27]. Montazeri et al. reported Cronbach's alpha of EPDS between 0.77 and 0.86 and its correlation coefficient as 0.80 during the postpartum period [28].

### Postpartum Specific Anxiety Questionnaire (PSAS)

The PSAS was invented by Davies et al. (2016) and its psychometric properties were confirmed. It is a valid and reliable tool for measuring postpartum anxiety. This 51-item questionnaire assesses four components of anxiety, including maternal competence and attachment anxieties, infant safety and welfare anxieties, practical infant care anxieties, and psychosocial adjustment to motherhood [29]. Owing to the high number of items, the 16-item version was considered the strongest version in terms of theory and psychometrics. The 16-item PSAS-RSF showed good psychometric properties and reliability. The PSAS-RSF is the first short instrument, validated to measure PPA. Based on the findings, it is theoretically significant and statistically strong, reliable and valid, which can be used up to 12 months after delivery. There are 4 items for each factor, and the options for the items are not at all (1), sometimes (2), often (3), and almost always (4), and the scores assigned to these options are 1 to 4, respectively. The total score range is between 16 and 64. This four-factor instrument had average to good reliability with McDonald's ω ranging from 0.65 to 0.8 and the overall scale had good reliability [30].

### Infant care behavior questionnaire

This 22-item questionnaire was compiled by Jamalivand et al. [31] in Iran based on the 4-point Likert scale from always (4), often (3), sometimes (2), and never (1) and its total score ranges between 22 and 88. Its validity was measured by the content validity index (CVI) and content validity ratio (CVR). Jamalivand et al. calculated CVI and CVR as 0.95 and 0.99, respectively. The Cronbach's alpha coefficient and Intra-class Correlation Coefficient (ICC) were obtained as 0.76 and 0.85, respectively.

### Barkin Index of Maternal Functioning (BIMF)

BIMF, developed by Barkin et al. (2007), was used to evaluate maternal functioning after childbirth. This 20-item questionnaire consists of seven domains, including self-care (2, 11, and 13), infant care (12 and 14), mother–child interaction (4, 5, and 15), psychological well-being of mother (1, 2, 3, 5, 7, 10, 11, 16, 18, and 20), social support (6, 8, and 9), management (7, 11, 13, 14, and 18), and adjustment (17 and 19) [32]. Each item is rated on a 7-point Likert scale ranging from 0 to 6 and the total score range is between 0 and 120. Higher score represents optimal maternal functioning. This questionnaire is used to measure maternal functioning during the 12 months after giving birth. Its important features include being patient-centered, taking into account all the fields related to postpartum functioning, containing favourable psychometric properties, and being applicable in both research and clinical methods. Mirghfourvand et al. psychometrically examined this tool in Iran, and Cronbach's alpha coefficient and ICC were calculated as 0.88 and 0.85, respectively, which indicates the validity and reliability of the tool [33].

### Violence questionnaire (HITS = Hurt, Insult, Threaten, and Scream)

This 4-item questionnaire focuses on physical and verbal violence on a 5-point Likert scale, ranging from 1 to 5 and the total score range is between 4 and 20. A score higher than 10 is considered positive, confirming the existence of violence. Asadi et al. psychometrically examined this tool in Iran and Cronbach's alpha coefficient and ICC was calculated as 0.78 and 0.86, respectively, indicating its validity and reliability [34].

### Data analysis

The data will be analyzed using SPSS version 24 software. The descriptive statistics will be used to describe socio-demographic and obstetric characteristics including the number (percent) for qualitative variables, the mean (standard deviation) for normal quantitative data, and the median (25th to 75th quartile) for abnormal quantitative data. The parametric tests will be used for normally distributed data and non-parametric tests will be employed for non-normally distributed data. ANCOVA test will be used by controlling the confounding factors, such as baseline score and stratification factor to compare quantitative variables, including postpartum depression, specific postpartum anxiety, maternal functioning, and experience of violence and infant care behavior between the intervention and control groups. The multivariable logistic regression will be applied for controlling the possible confounding variables, including the stratification factor to compare the qualitative variables, including maternal health problems between the intervention and control groups.

### Ethical considerations

The present study has been approved by the Ethics Committee of Tabriz University of Medical Sciences (Ethical code: IR.TBZMED.REC.1401.661) and has been registered in the Iranian Registry of Clinical Trials (IRCT20120718010324N76). The written informed consent will be obtained from all participants during the study. Participants will be assured of the confidentiality of their information and names in the results report and they will be allowed to leave the study at any stage of the study if they wish.

## Discussion

After childbirth, women and their babies are vulnerable to a variety of physiological, psychological, and social problems, especially within 24 h after birth, which is a great threat to the health of the mother and baby. On the other hand, there has been a global change in the mother and child health program from focusing only on survival to the transformation and flourishing of programs and the provision of high-quality care, as the change is in line with the third goal of SDGs. Further, good quality clinical care and improvement of communication, education, information, and respectful behavior of care providers are essential aspects of the care for women. A combination of these factors is necessary to keep mothers and their babies safe and deal with threats. In the framework of primary care of women and newborns after delivery, evidence-based guidelines can reduce mid- and long-term complications by improving care, influencing the policy-making levels by providing recommendations in the field of clinical management, and help to the care departments and professions by ensuring the continuity and uniformity of care.

Clinical guidelines provide a pathway for medical service providers to access evidence-based recommendations with critical evaluation for patient care. High-quality clinical guidelines are crucial as they are based on rigorous evidence, careful interpretations of the evidence, and systematic methods of guideline development. Additionally, these guidelines are influenced by professional bodies, patient preferences, cultural and socioeconomic factors, and are consistent and appropriate for use in any resource setting. The World Health Organization (WHO) recommends adapting and implementing these guidelines to the specific needs of different countries in order to ensure respectful, individualized, person-centered care at every interaction, in accordance with a human rights-based approach. By doing so, healthcare providers can deliver the best possible care to their patients, regardless of their location or background (1).

Considering that there is a growing consensus among public health professionals that midwifery care has a fundamental contribution to high-quality services for mothers and infants, this study aims to localize guideline recommendations and adapt them to national guidelines in the field of postpartum care. It is expected that by applying the adapted evidence-based recommendations in care and measuring their consequences, this study can represent an important step towards improving the quality of care and enhancing the health of mothers and newborns.

Due to limitations in implementing recommendations related to the health system, the research team has decided to focus only on the recommendations related to mothers, infants, and families in this study.

### Supplementary Information

**Additional file 1.** Search strategy.

## Data Availability

Not applicable.

## Abbreviations

WHO: World Health Organization
SDGs: Sustainable development goals
ADAPTE: Group name for adaption a Clinical guideline
AGREE: Appraisal of Guidelines for Research and Evaluation
