# Supplementary material for: Adaptation and implementation of clinical guidelines on maternal and newborn postnatal care in Iran: study protocol
Source: Reprod Health. 2023 Sep 12;20:135. doi: 10.1186/s12978-023-01682-0 (PMC10496192; doi:10.1186/s12978-023-01682-0)
Supplement: Supplementary file 1 — Additional file 1. Search strategy. [file 12978_2023_1682_MOESM1_ESM.docx]

**Search Strategy**

**PubMed**

| **Search number** | **Query** | **Filters** | **Results** |
| --- | --- | --- | --- |
| 1 | (postpartum care) OR (postpartum period) OR (postnatal period) OR (postnatal care) OR (puerperium) OR (postpartum programs) | | 146,559 |
| 2 | (guideline) OR (guidance) OR (recommendation) OR (consensus) OR (best practice) OR (statement) | | 1,560,110 |
| 3 | ((postpartum care) OR (postpartum period) OR (postnatal period) OR (postnatal care) OR (puerperium) OR (postpartum programs)) AND ((guideline) OR (guidance) OR (recommendation) OR (consensus) OR (best practice) OR (statement)) | | 11,919 |
| 6 | ("postnatal care"[MeSH Terms]) OR (postnatal care[Title/Abstract]) OR ("postpartum period"[MeSH Terms]) OR (postpartum period[Title/Abstract]) OR (postnatal period[Title/Abstract]) OR (puerperium[Title/Abstract]) OR (postpartum program*[Title/Abstract]) | | 99,360 |
| 7 | (((guideline[Title/Abstract]) OR (guidance[Title/Abstract]) OR (recommendation*[Title/Abstract]) OR (consensus[Title/Abstract]) OR (best practice[Title/Abstract]) OR (statement*[Title/Abstract])) OR ("guideline"[Publication Type])) OR ("consensus"[MeSH Terms]) | | 833,773 |
| 8 | (("postnatal care"[MeSH Terms]) OR (postnatal care[Title/Abstract]) OR ("postpartum period"[MeSH Terms]) OR (postpartum period[Title/Abstract]) OR (postnatal period[Title/Abstract]) OR (puerperium[Title/Abstract]) OR (postpartum program*[Title/Abstract])) AND ((((guideline[Title/Abstract]) OR (guidance[Title/Abstract]) OR (recommendation*[Title/Abstract]) OR (consensus[Title/Abstract]) OR (best practice[Title/Abstract]) OR (statement*[Title/Abstract])) OR ("guideline"[Publication Type])) OR ("consensus"[MeSH Terms])) | | 3,752 |
| 9 | (("postnatal care"[MeSH Terms]) OR (postnatal care[Title/Abstract]) OR ("postpartum period"[MeSH Terms]) OR (postpartum period[Title/Abstract]) OR (postnatal period[Title/Abstract]) OR (puerperium[Title/Abstract]) OR (postpartum program*[Title/Abstract])) AND ((((guideline[Title/Abstract]) OR (guidance[Title/Abstract]) OR (recommendation*[Title/Abstract]) OR (consensus[Title/Abstract]) OR (best practice[Title/Abstract]) OR (statement*[Title/Abstract])) OR ("guideline"[Publication Type])) OR ("consensus"[MeSH Terms])) | Guideline | 234 |
| 10 | (((Assessment) OR (Screening)) OR (prevention)) OR (education) | | 12,426,372 |
| 11 | (((("diagnosis"[Subheading]) OR ("prevention and control"[Subheading])) OR ("education"[Subheading])) OR ("education"[MeSH Terms])) OR ((Assessment[Title/Abstract]) OR (Screening[Title/Abstract]) OR (prevention[Title/Abstract]) OR (education[Title/Abstract])) | | 8,072,738 |
| 12 | ((((("diagnosis"[Subheading]) OR ("prevention and control"[Subheading])) OR ("education"[Subheading])) OR ("education"[MeSH Terms])) OR ((Assessment[Title/Abstract]) OR (Screening[Title/Abstract]) OR (prevention[Title/Abstract]) OR (education[Title/Abstract]))) AND ((("postnatal care"[MeSH Terms]) OR (postnatal care[Title/Abstract]) OR ("postpartum period"[MeSH Terms]) OR (postpartum period[Title/Abstract]) OR (postnatal period[Title/Abstract]) OR (puerperium[Title/Abstract]) OR (postpartum program*[Title/Abstract])) AND ((((guideline[Title/Abstract]) OR (guidance[Title/Abstract]) OR (recommendation*[Title/Abstract]) OR (consensus[Title/Abstract]) OR (best practice[Title/Abstract]) OR (statement*[Title/Abstract])) OR ("guideline"[Publication Type])) OR ("consensus"[MeSH Terms]))) | | 1,935 |
| 13 | ((((("diagnosis"[Subheading]) OR ("prevention and control"[Subheading])) OR ("education"[Subheading])) OR ("education"[MeSH Terms])) OR ((Assessment[Title/Abstract]) OR (Screening[Title/Abstract]) OR (prevention[Title/Abstract]) OR (education[Title/Abstract]))) AND ((("postnatal care"[MeSH Terms]) OR (postnatal care[Title/Abstract]) OR ("postpartum period"[MeSH Terms]) OR (postpartum period[Title/Abstract]) OR (postnatal period[Title/Abstract]) OR (puerperium[Title/Abstract]) OR (postpartum program*[Title/Abstract])) AND ((((guideline[Title/Abstract]) OR (guidance[Title/Abstract]) OR (recommendation*[Title/Abstract]) OR (consensus[Title/Abstract]) OR (best practice[Title/Abstract]) OR (statement*[Title/Abstract])) OR ("guideline"[Publication Type])) OR ("consensus"[MeSH Terms]))) | Guideline | 162 |
